# Effects of 1 year of exercise training versus combined exercise training and weight loss on body composition, low-grade inflammation and lipids in overweight patients with coronary artery disease: a randomized trial

**DOI:** 10.1186/s12933-019-0934-x

**Published:** 2019-10-01

**Authors:** Lene Rørholm Pedersen, Rasmus Huan Olsen, Christian Anholm, Arne Astrup, Jesper Eugen-Olsen, Mogens Fenger, Lene Simonsen, Rosemary L. Walzem, Steen Bendix Haugaard, Eva Prescott

**Affiliations:** 1 Department of Cardiology, Bispebjerg University Hospital, University of Copenhagen, Building 67, 1st, Bispebjerg Bakke 23, 2400 Copenhagen, NW Denmark; 2 Department of Cardiology, University Hospital of Zealand, Roskilde, University of Copenhagen, Copenhagen, Denmark; 3 Department of Internal Medicine, Glostrup University Hospital, University of Copenhagen, Copenhagen, Denmark; 4 Department of Nutrition, Exercise and Sports, University of Copenhagen, Copenhagen, Denmark; 5 Clinical Research Centre, Hvidovre University Hospital, University of Copenhagen, Copenhagen, Denmark; 6 Department of Medical Biochemistry, Genetics and Molecular Biochemistry, Hvidovre University Hospital, University of Copenhagen, Copenhagen, Denmark; 7 Department of Clinical Physiology and Nuclear Medicine, Bispebjerg University Hospital, University of Copenhagen, Copenhagen, Denmark; 8 Faculty of Nutrition, Texas A & M University, College Station, TX USA; 9 Department of Internal Medicine, Amager and Hvidovre University Hospital, University of Copenhagen, Copenhagen, Denmark

**Keywords:** Aerobic interval training, Weight loss, Secondary prevention, Coronary artery disease, Dyslipidaemia, Low-grade inflammation, Dyslipidaemia

## Abstract

**Background:**

Dyslipidaemia and low-grade inflammation are central in atherogenesis and linked to overweight and physical inactivity. Lifestyle changes are important in secondary prevention of coronary artery disease (CAD). We compared the effects of combined weight loss and interval training with interval training alone on physical fitness, body composition, dyslipidaemia and low-grade inflammation in overweight, sedentary participants with CAD.

**Methods:**

Seventy CAD patients, BMI 28–40 kg/m^2^ and age 45–75 years were randomised to (1) 12 weeks’ aerobic interval training (AIT) at 90% of peak heart rate three times/week followed by 40 weeks’ AIT twice weekly or (2) a low energy diet (LED) (800–1000 kcal/day) for 8–10 weeks followed by 40 weeks’ weight maintenance including AIT twice weekly and a high-protein/low-glycaemic load diet. Effects of the intervention were evaluated by physical fitness, body weight and composition. Dyslipidaemia was described using both biochemical analysis of lipid concentrations and lipoprotein particle subclass distribution determined by density profiling. Low-grade inflammation was determined by C-reactive protein, soluble urokinase-type plasminogen activator receptor and tumour necrosis factor α. Effects on continuous outcomes were tested by mixed-models analysis.

**Results:**

Twenty-six (74%) AIT and 29 (83%) LED + AIT participants completed the study. At baseline subject included 43 (78%) men; subjects averages were: age 63 years (6.2), body weight 95.9 kg (12.2) and VO_2_peak 20.7 mL O_2_/kg/min (4.9). Forty-six (84%) had pre-diabetes (i.e. impaired fasting glucose and/or impaired glucose tolerance). LED + AIT reduced body weight by 7.2 kg (− 8.4; − 6.1) and waist circumference by 6.6 cm (− 7.7; − 5.5) compared to 1.7 kg (− 0.7; − 2.6) and 3.3 cm (− 5.1; − 1.5) after AIT (within-group p < 0.001, between-group p < 0.001 and p = 0.018, respectively). Treatments caused similar changes in VO_2_peak and lowering of total cholesterol, triglycerides, non-HDL cholesterol and low-grade inflammation. A shift toward larger HDL particles was seen following LED + AIT while AIT elicited no change.

**Conclusions:**

Both interventions were feasible. Both groups obtained improvements in VO_2_peak, serum-lipids and inflammation with superior weight loss and greater central fat loss following LED + AIT. Combined LED induced weight loss and exercise can be recommended to CAD patients.

*Trial registration* NCT01724567, November 12, 2012, retrospectively registered (enrolment ended in April 2013).

## Introduction

Physical inactivity and obesity, abdominal obesity in particular, are known risk factors in coronary artery disease (CAD). Lifestyle interventions to reduce body weight and increase physical activity are cornerstones of secondary CAD prevention [1–3] Exercise-based cardiac rehabilitation programmes lower hospital admissions, total and cardiovascular mortality [4]. The EUROASPIRE IV study, evaluating the secondary prevention effort in CAD in Europe from 2012 to 2013, showed that 82.1% of CAD patients were overweight or obese (BMI > 25 kg/m^2^) and 58.2% were centrally obese based on waist circumference. These numbers have gradually increased compared to previous EUROASPIRE cohorts dating back to 1995–1996. In the same population 60% of the patients reported little or no physical activity and poor metabolic risk factor control concerning dyslipidaemia and type 2 diabetes was described [5]. Thus, effective and sustainable cardiac rehabilitation strategies are needed.

Dyslipidaemia and increased levels of low-grade inflammation are linked to obesity and physical inactivity [1, 6, 7] and related to excess cardiovascular risk in healthy population [8–12] and to a poor prognosis in patients with CAD [13–15]. Both exercise and weight loss interventions have been shown to improve dyslipidaemia [16–19] and reduce low-grade inflammation [20–22]. Atherogenic lipoproteins in the intima and media of the arterial wall elicit an inflammatory response that initiate and promote atherosclerotic plaque [6]. The amount of LDL-C and HDL-C in blood is used to monitor cholesterol-lowering treatment [1]; however, lipoprotein density profiling to measure lipoprotein density subclasses is used in the current report to give a more precise risk estimation by taking into account the contribution of small, dense atherogenic lipoprotein particles [23]. In the present paper low-grade inflammation is described using C-reactive protein (CRP), tumour necrosis factor α (TNFα) and soluble urokinase-type plasminogen activator receptor (suPAR). CRP is a well-known marker of low-grade inflammation [12] while TNFα is important in the link between obesity, sedentary behaviour, atherosclerosis and insulin resistance [6]. The more recently described suPAR is believed to play a role in the development of the unstable plaque [24].

A recent systematic review showed that aerobe exercise training for 12 weeks or more improved body composition, metabolic outcome and physical fitness in individuals with metabolic syndrome [25] and in patients with type 2 diabetes aerobic exercise at high intensities improves physical fitness and glycaemic control [26]. A 12-week study comparing diet-induced weight loss, exercise-induced weight loss and exercise without weight loss in healthy overweight populations found that all interventions improved body composition. The greatest body composition improvement was seen in the weight loss groups, while physical fitness was improved in the exercise group [27]. This is in accordance with previously reported results in the present trial demonstrating that a rapid 10% weight loss using a low energy diet (LED) was superior to a 12–week aerobic interval training (AIT) programme in reducing body weight, body fat mass, waist circumference and lipid atherogenicity. Low-grade inflammation was largely unchanged in both groups. Exercise training was superior to weight loss alone in improving physical fitness [28, 29].

Cardiac rehabilitation programmes are often short-term interventions and sustaining the effects obtained is a major challenge. A long-term comparison of weight loss induced by exercise, caloric restriction or a combined intervention in a healthy population showed that a combination of weight loss and exercise provides greater improvement in physical function than either intervention alone [30]. A 2009 study in which 74 participants with CAD were randomised to either 5 months of high-calorie-expenditure exercise or a less intense standard cardiac rehabilitation exercise found that intense exercise caused a greater weight loss and a more favourable cardiometabolic risk profile [31]. However, there is a paucity of short- and long-term trials addressing lifestyle intervention and maintenance programmes in CAD patients [1, 32].

To address this gap we wish to describe the effect of a long-term 1-year intervention. The current paper details effects on physical fitness and metabolic risk described using body composition, dyslipidaemia and low-grade inflammation following the entire 1-year intervention in the randomised CUT IT trial. The CUT IT trial compared the effects of a combined weight loss and aerobic interval training (AIT) programme to AIT alone in a randomised design. We hypothesized that a strategy of weight loss followed by AIT would be superior to AIT alone to achieve long-term success.

## Methods

### Study design

Seventy participants were consecutively enrolled and randomised 1:1 to either AIT or low energy diet weight loss and AIT (LED + AIT). Inclusion criteria were stable CAD diagnosed > 6 months prior to inclusion, age 45–75 years and BMI 28–40 kg/m^2^. Exclusion criteria were known diabetes or diabetes diagnosed at the screening visit, other severe heart disease (i.e. heart failure EF < 35%, severe or moderate valve disease, main stem stenosis and arrhythmias or ischaemia revealed by the cardiopulmonary exercise test and 2. or 3. degree AV block not protected by a pacemaker) or severe co-morbidity (i.e. chronic pulmonary disease, active cancer or severe kidney failure). Furthermore, candidates who participated in organised sports more than twice weekly or had experienced a significant weight loss or gain (> 5%) more than 3 months prior to the screening visit were excluded.

1. AIT: 12 weeks’ supervised AIT three times weekly followed by 40 weeks’ AIT twice weekly. Each exercise session was preceded by a 10-minute warm-up on stairs or an exercise bike followed by high intensity interval training on an exercise bike. The high intensity intervals (85–90% of VO_2_peak, Borg scale 17–18) lasted between 1 and 4 min, to achieve a total of 16 min, separated by active pauses (65–70% of VO_2_peak) of 1- and 3-min duration. The total duration of each training session was 48 min including the warm-up. Physiotherapists with experience in cardiac rehabilitation instructed the participants and supervised all training session. Training intensity was monitored with heart rate monitors and perceived exertion using the Borg Scale [33]. Additional file 1: Figure S1 shows a heart rate curve from an exercise session to illustrate the exercise intervention.
2. LED + AIT: 8–10 weeks’ LED (800–1000 kcal/day, the Cambridge Weight Plan, Northants, UK) followed by 2–4 weeks’ transition to a maintenance diet to avoid examining the participants in a catabolic state. The last 40 weeks included the maintenance diet and AIT twice weekly. The maintenance diet was a low glycaemic load diet achieved by slightly higher protein content and focus on low glycaemic index carbohydrates as described in the DIOGenes study [34]. The LED and the maintenance diet were supervised by experienced dieticians.

A more comprehensive description of study design, population and interventions was published previously [35].

### Body weight and composition

Body weight, hip and waist circumference were determined in the morning after a 10-h fast. Waist circumference was measured halfway between the lower rib and the iliac crest and hip circumference at the maximal gluteal protuberance and calculated as an average of two consecutive measurements. Fat mass and fat free mass were estimated using a whole-body dual X-ray absorptiometry scan (Lunar DPX-IQ, GE Lunar Corp, Madison, WI, USA).

### Physical fitness and self-reported physical activity

**Fig. 1:**
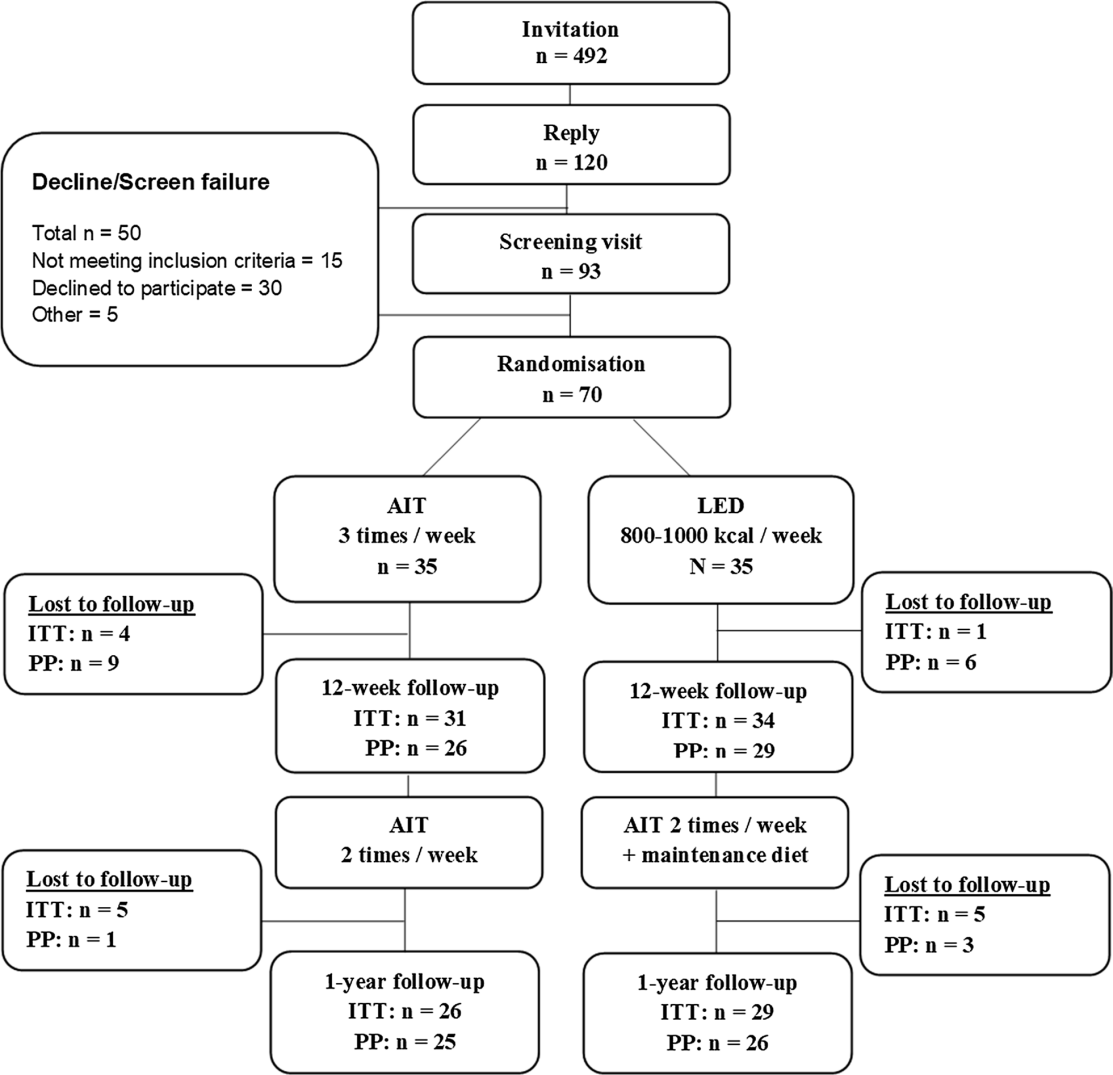
Inclusion and course of the study. AIT, Aerobic interval training, LED, Low energy diet. ITT: Intention-to-treat including all participants attending the follow-up. PP: per protocol. PP 12-weeks: including all participants who met per protocol criteria. PP 1 year: including all participants who completed the 12-week intervention per protocol and attended the one-year follow-up

VO_2_peak was assessed by a stepwise cardiopulmonary exercise test (CPET) using a bicycle ergometer (Via Sprint 150 P, Ergoline, Bitz, Germany) with breath-by-breath gas exchange measurements (Jaeger, MasterScreenCPX, Cardinal Health, Wurzburg, Germany). The participants completed a CPET at the screening visit (Fig. 1) to obtain familiarisation to the test. Criteria for VO_2_peak were levelling off of VO_2_ despite increasing workload or peak respiratory exchange ratio (peakRER) > 1.05. VO_2_peak and peakRER were calculated as the mean of the six highest consecutive five-second measurements of VO_2_ and VO_2_/VCO_2_ before exercise termination. VO_2_peak was expressed as: VO_2_peak_total_ (mL/min), VO_2_peak_bw_ (mL/kg body weight/min) and, to account for changes in body composition, VO_2_peak_ffm_ (mL/kg fat free mass^0.67^/min) [36]. Peak heart rate determined during the CPET was used to monitor the exercise sessions.

The international physical activity questionnaire short form (iPAQ-SF) [37] was used at all visits and self-reported physical activity duration and intensity was used to calculate weekly physical activity energy expenditure in kilocalories according to the IPAQ Research Committee recommendations [38]. The participants were asked to include the supervised exercise sessions.

### Lipids and density profiling

Blood samples were taken in the morning after a 10-hour overnight fast. Plasma lipids (i.e. total cholesterol, high-density lipoprotein cholesterol (HDL-C) and triglycerides) were analysed immediately at the hospital laboratory. Low-density lipoprotein cholesterol (LDL-C) was calculated using Friedewald’s equation (LDL-C = total cholesterol − HDL-C + 0.45 × triglycerides). One subject was excluded due to triglycerides > 4.5 mmol/L [1]. Total cholesterol/HDL-C-ratio and non-HDL-C = total cholesterol − HDL-C were calculated. To further describe the atherogenicity of the lipoproteins additional information on lipoprotein particle density and subclass distribution were obtained using isopycnic density profiling of lipoproteins pre-stained with a lipophilic fluorescent probe as we have described previously [28]. In that analysis lipoprotein amount is measured by area under the curve (AUC). LDL and HDL particle size were estimated based in the average particle size for each subfraction and the percentage each subfraction constituted of total LDL or HDL, respectively.

### Inflammatory markers

Tumour necrosis factor α (TNFα) was determined using an enzyme-linked immunosorbent assay (ELISA, DRG instruments Marburg, Germany). Soluble urokinase plasminogen activator receptor (SuPAR) was analysed using suPARnostic^®^ ELISA (ViroGates, Copenhagen, Denmark). C-reactive protein (CRP) was determined using a high-sensitivity assay ELISA with a lower detection limit of 0.2 mg/L. All kits were used according to the manufacturer instructions. For intra- and inter-assay variation please see Ref. [28].

### Blood pressure

Blood pressure was measured in the morning > 18 h after the latest exercise session in the supine position after 10 min’ rest as an average of the last two of three consecutive measurements on the dominant arm using an oscillometric blood pressure monitor (CARESCAPE V100, GE Healthcare, Horten, Norway).

### Hospital Anxiety and Depression Scale (HADS) and symptoms

Anxiety and depression were evaluated by the self-assessment Hospital Anxiety and Depression Scale (HADS) separated into anxiety (HADS-A) and depression (HADS-D) sub-scales [39].

### Intention-to-treat and per protocol analyses

All participants were invited and encouraged to attend the 12-week follow-up, the 40-week maintenance period and 1-year follow-up independently of adherence to protocol. The main analyses presented are intention-to-treat including all patients who attended the baseline visit and the two follow-up visits at 12 and 52 weeks. Per protocol criteria of the 12-week intervention in the LED + AIT group were ≥ 5% weight loss whereas the AIT group required overall training attendance ≥ 60% and attendance ≥ 50% the last 2 weeks of the intervention. Analyses including all participants who completed the 12-week intervention per protocol and attended the 1-year follow-up are presented in Additional file 1.

### Statistical analyses

The group sample size of 26 participants was calculated based on the primary end point of coronary velocity flow reserve; a further allowed drop-out rate of up to 25% brought final study size to 70 participants [35]. At baseline categorical data are presented as number (percentage), normally distributed data as mean (SD) and non-normally distributed data as median (inter-quartile (IQ) range). Baseline comparisons were made using χ^2^ or Fischer’s exact test, unpaired t-test and Wilcoxon-rank test, respectively. Effects on continuous outcomes were tested by mixed-models with subject id as a random factor. Between-group differences were tested using models with group, visit and group*visit interaction as fixed factors, whereas within-group changes were tested in separate models for each group. Logarithmic transformation was performed on TNFα, CRP and suPAR values ensuring a normal distribution. The significance level was set to *p *< 0.05. Stata 13.1 software (StataCorp, College Station, TX, USA) was used for all analyses.

## Results

### Population

A total of 70 participants were randomised, the 55 (79%) that completed the study are included in these intention-to-treat analyses (Fig. 1). At baseline, 39 (76%) were male, mean age was 63 years (SD 6.2), median BMI was 31.4 (IQ-range 29.8; 33.5). All participants were non-diabetic; however, 46 (84%) had pre-diabetes (i.e. impaired fasting glucose and/or impaired glucose tolerance). Additional baseline data are presented in Table 1. Overall, participants were asymptomatic, well-controlled regarding blood pressure and lipids and contemporarily treated with platelet inhibitors, statins, ACE-inhibitors/angiotensin receptor blockers and beta-blockers (Table 1). Baseline characteristics of the per protocol population did not differ from the intention to treat population (Additional file 1: Table S1). Participants who had alterations made to their statin treatment were excluded from analyses of lipids and inflammatory markers. At 12 weeks this applied to one participant in the AIT group and at 1 year one participant undergoing LED + AIT.

**Table 1:** Baseline characteristics

	AIT (n = 26)	LED + AIT (n = 29)	p
Male	22 (85%)	21 (72%)	0.34
Age	62.3 (5.7)	63.8 (6.7)	0.38
VO_2_peak_ffm_ (mL/kg ffm^0.67^/min)	124 (24)	126 (25)	0.83
VO_2_peak_bw_ (mL/kg/min)	20.8 (4.9)	20.6 (5.0)	0.85
Body weight (kg)	96.2 (13.8)	95.5 (10.7)	0.85
Body mass index (kg/m^2^)	31.5 (29.6; 33.5)	31.3 (29.9; 33.7)	0.85
Body fat mass (kg)	32.6 (7.6)	34.6 (8.0)	0.36
Waist circumference (cm)	110 (10)	108 (7)	0.36
Hip circumference (cm)	111 (8)	110 (7)	0.80
Systolic blood pressure (mmHg)	126 (13)	127 (15)	0.78
Diastolic blood pressure (mmHg)	74 (9.2)	71 (7.7)	0.18
Total cholesterol (mmol/L)	4.3 (0.8)	4.1 (0.7)	0.23
Left ventricular ejection fraction (%)	53 (8)	53 (7)	0.97
Pre-diabetes	21 (81%)	25 (86%)	0.59
Ischaemic aetiology and treatment			
Myocardial infarction	8 (31%)	22 (76%)	< 0.001
Percutaneous coronary intervention	17 (65%)	23 (79%)	0.25
Coronary artery bypass graft	6 (23%)	6 (21%)	0.83
Prior cardiac rehabilitation	24 (92%)	21 (72%)	0.06
CCS-class			
0	21 (81%)	23 (79%)	
I	5 (19%)	6 (21%)	0.89
NYHA-class			
I	21 (81%)	21 (72%)	
II	5 (19%)	7 (24%)	0.87
III	0 (0%)	1 (3%)	
Medication			
ACE-I/ARB	15 (58%)	21 (72%)	0.57
Acetylsalicylic acid	23 (88%)	26 (90%)	1.00
Beta blocker	12 (46%)	16 (55%)	0.50
Calcium antagonist	6 (23%)	10 (34%)	0.39
Statin	25 (96%)	28 (97%)	1.00
Other cholesterol-lowering drug	5 (19%)	2 (7%)	0.24

Baseline characteristics intention-to-treat population. Categorical data: number (%), normally distributed data: mean (SD), non-normally distributed data: median (IQ-range). p-values: between-group differences

AIT, aerobic interval training; LED, low energy diet; VO_2_peak_bw_, peak aerobic capacity corrected for body weight; VO_2_peak_ffm_, peak aerobic capacity corrected for fat free mass, CCS, Canadian Cardiovascular Society; NYHA, New York Heart Association; ACE-I, angiotensin converting enzyme inhibitor; ARB, angiotensin receptor blocker

The main results presented are intention-to-treat analyses of the changes from baseline to 1 year. Results from per protocol analyses are presented in Additional file 1 and differences from intention-to-treat outcomes noted in the text. Results from the 12-week intervention are briefly mentioned where relevant.

### Weight loss and body composition

The participants in the LED + AIT group lost 10.6% of body weight (p < 0.001) during the first 12 weeks’ intervention while the AIT group lost a small but significant 1.6% of body weight (p = 0.002). Both groups experienced concomitant decreases in waist circumference and body fat mass; albeit, largest after LED (between-group p < 0.001) [29]. Despite a significant increase in body weight, body fat mass, waist circumference and waist/hip-ratio in the LED + AIT group during the 40-week maintenance period body composition remained significantly improved compared to baseline at 1 year. A non-significant 0.5 kg (p = 0.060) decrease in fat free mass was seen in the intention to treat analysis. Per protocol analysis showed a 0.6 kg decrease in fat free mass (p = 0.049) at 1 year in LED + AIT. However, from 12 weeks to 1 year fat free mass in the LED + AIT group increased a non-significant 0.3 kg in both intention to treat and per protocol analyses (Table 2, Additional file 1: Table S2). The AIT group maintained the effects on body composition obtained during the first 12 week. After 1 year LED + AIT was still superior to AIT alone with regards to reducing body weight, body fat mass and waist circumference (Table 2). Per protocol analysis only differed from intention to treat with regard to fat free mass in the LED + AIT group (Additional file 1: Table S2).

**Table 2:** Body composition and physical fitness

	AIT (n = 26)					LED + AIT (n = 29)					Between-group 1 year^a^	p
	Baseline	Change baseline to 1 year	p	Change 12 weeks to 1 year	p	Baseline	Change baseline to 1 year	p	Change 12 weeks to 1 year	p		
Body composition												
Weight (kg)	96.2 (13.8)	− 1.7 (− 0.7; − 2.6)	< 0.001	− 0.4 (− 1.4; 0.6)	0.406	95.6 (10.7)	− 7.2 (− 8.4; − 6.1)	< 0.0 01	2.5 (1.3; 3.6)	< 0.001	5.6 (4.1; 7.1)	< 0.001
Body mass index (kg/m^2^)	32.1 (3.2)	− 0.6 (− 0.9; − 0.2)	0.001	− 0.1 (− 0.5; 0.2)	0.408	32.2 (3.1)	− 2.5 (− 2.9; − 2.1)	< 0.001	0.8 (0.4; 1.2)	< 0.001	1.90 (1.37; 2.42)	< 0.001
Body fat mass (kg)	32.6 (7.6)	− 1.9 (− 2.8; − 1.0)	< 0.001	− 0.3 (− 1.2; 0,5)	0.448	34.6 (8.0)	− 6.6 (− 7.7; − 5.5)	< 0.001	1.9 (0.8; 3.1)	< 0.001	4.7 (3.2; 6.2)	< 0.001
Body fat %	34.6 (6.5)	− 1.5 (− 2.4; − 0.7)	< 0.001	0.2 (− 1.0; 0.6)	0.618	36.7 (6.8)	− 4.7 (− 5.8; − 3.7)	< 0.001	1.6 (0.5; 2.7)	0.003	3.2 (1.8; 4.6)	< 0.001
Fat free mass (kg)	62.9 (9.9)	0.2 (− 0.5; 0.9)	0.564	− 0.3 (− 0.9; 0.4)	0.430	60.4 (8.8)	− 0.5 (− 1.1; 0.02)	0.060	0.3 (− 0.2; 0.9)	0.240	0.7 (− 0.1; 1.6)	0.096
Waist (cm)	109.9 (9.5)	− 3.3 (− 5.1; − 1.5)	< 0.001	− 0.4 (− 2.2; 1.4)	0.696	107.7 (7.1)	− 6.6 (− 8.6; − 4.6)	< 0.001	3.2 (1.2; 5.2)	0.002	3.3 (0.6; 6.1)	0.018
Hip (cm)	110.8 (8.0)	− 4.0 (− 5.1; − 2.9)	< 0.001	− 1.7 (− 2.8; − 0.6)	0.003	110.3 (7.5)	− 6.0 (− 7.4; − 4.7)	< 0.001	0.9 (− 0.4; 2.2)	0.201	2.0 (0.3; 3.8)	0.025
Waist/hip– ratio	0.99 (0.1)	0.005 (− 0.01; 0.02)	0.512	0.01 (− 0.003; 0.03)	0.116	0.98 (0.1)	− 0.01 (− 0.03; 0.01)	0.397	0.02 (0.01; 0.02)	0.008	0.01 (− 0.01; 0.04)	0.295
Physical fitness												
VO_2_peak_total_ (mL/min)	1997 (463)	95 (− 30; 221)	0.135	− 123 (− 249; 2)	0.053	1981 (533)	132 (37; 227)	0.007	196 (102; 292)	< 0.001	− 36 (− 192; 119)	0.645
VO_2_peak_kg_ (mL/kg/min)	20.8 (4.9)	1.5 (0.2; 2.9)	0.027	− 1.0 (− 2.3; 0.4)	0.150	20.6 (5.2)	3.1 (2.9; 4.2)	< 0.001	1.5 (0.4; 2.7)	0.007	− 1.6 (− 3.3; 0.2)	0.077
VO_2_peak_ffm_ (mL/kg fat free mass^0.67^/min)	124.0 (24.3)	5.9 (− 1.9; 13.7)	0.138	− 7.4 (− 15.2; 0.4)	0.065	125.5 (25.2)	8.7 (2.8; 14.6)	0.004	11.9 (6.0; 17.9)	< 0.001	− 2.8 (− 12.4; 6.9)	0.575
Peak RER	1.19 (0.08)	0.0002 (− 0.03; 0.03)	0.989	0.01 (− 0.02; 0.04)	0.602	1.18 (0.09)	0.06 (− 0.02; 0.09)	< 0.001	0.00001 (− 0.03; 0.03)	0.999	− 0.05 (− 0.1; − 0.01)	0.018
Max workload (W)	158 (42)	13 (2; 23)	0.017	− 7.6 (− 18; 3)	0.155	155 (48)	15 (9; 22)	< 0.001	15 (9; 22)	< 0.001	− 2.5 (− 14.4; 9.5)	0.687
Peak heart rate	137 (23)	1 (− 4; 7)	0.638	1 (− 6; 5)	0.826	135 (22)	9 (4; 13)	< 0.001	9 (− 4; 14)	< 0.001	− 7.4 (− 14.5; − 0.3)	0.040

Intention-to-treat analysis. Baseline: mean (SD) Baseline data are presented as mean (SD), within- and between-group differences are presented with 95% CI. AIT, Aerobic interval training, LED, Low energy diet, VO_2_peak_total_, Total peak aerobic capacity, VO_2_peak_bw_, Peak aerobic capacity corrected for body weight, VO_2_peak_ffm_, Peak aerobic capacity corrected for fat free mass, CPET, Cardiopulmonary exercise test, RER, respiratory exchange ratio

^a^Between-group difference at 1 year corrected for baseline difference

### Physical activity

During the first 12 weeks VO_2_peak_ffm_ improved by 11.4% and workload by 10.4% in the AIT group (p < 0.002) while both remained unchanged after LED [29]. Median training attendance during the last 40 weeks was 63% (IQ-range 49%; 82%) and 50% (IQ-range 0%; 70%) in the AIT and LED + AIT group, respectively. During the maintenance period there was a small but non-significant decrease in all measures of physical fitness in the AIT group such that only the increase in VO_2_peak_bw_ and CPET workload remained statistically improved at 1 year (p < 0.027 and 0.017, respectively). In the LED + AIT group a significant improvement in physical fitness was obtained with regard to all measures of physical fitness compared to baseline (Table 2). Per protocol analyses did not differ from the intention to treat analyses (Additional file 1: Table S2). After 1 year there was no difference between the groups in physical fitness variables. One participant in each group did not complete the CPET due to claustrophobia or hip pain. One participant in the LED + AIT group did not attend the CPET at 1 year due to recent knee surgery.

At baseline the AIT group seemed to have a higher physical activity level than the LED + AIT group (6204 (4069) kcal/week vs. 4381 (2794) kcal/week though the difference was not significant (p = 0.345). At 1 year the weekly energy expenditure increased by 2359 kcal/week (95% CI 528; 4191, p = 0.012) in the LED + AIT group and by 1408 kcal/week (95% CI − 2726; 5542, p = 0.504) in the AIT group. These changes resulted in similar weekly energy expenditures in the two groups at 1 year (p = 0.656).

### Lipids and lipoprotein subgroups

The 12-week intervention caused a significant decrease in chemically determined total cholesterol, non-HDL-C, total cholesterol/HDL-C ratio and triglycerides in both groups with no significant between-group differences. No changes were seen in HDL-C [29]. After 1 year a significant decrease was seen in triglycerides, total and non-HDL-C cholesterol in both groups while LDL-C and HDL-C remained unchanged. A decrease in total cholesterol/HDL-C-ratio was only seen after LED + AIT. There was no difference between the intention to treat and per protocol analyses (Additional file 1: Tables S3 and S4).

**Fig. 2:**
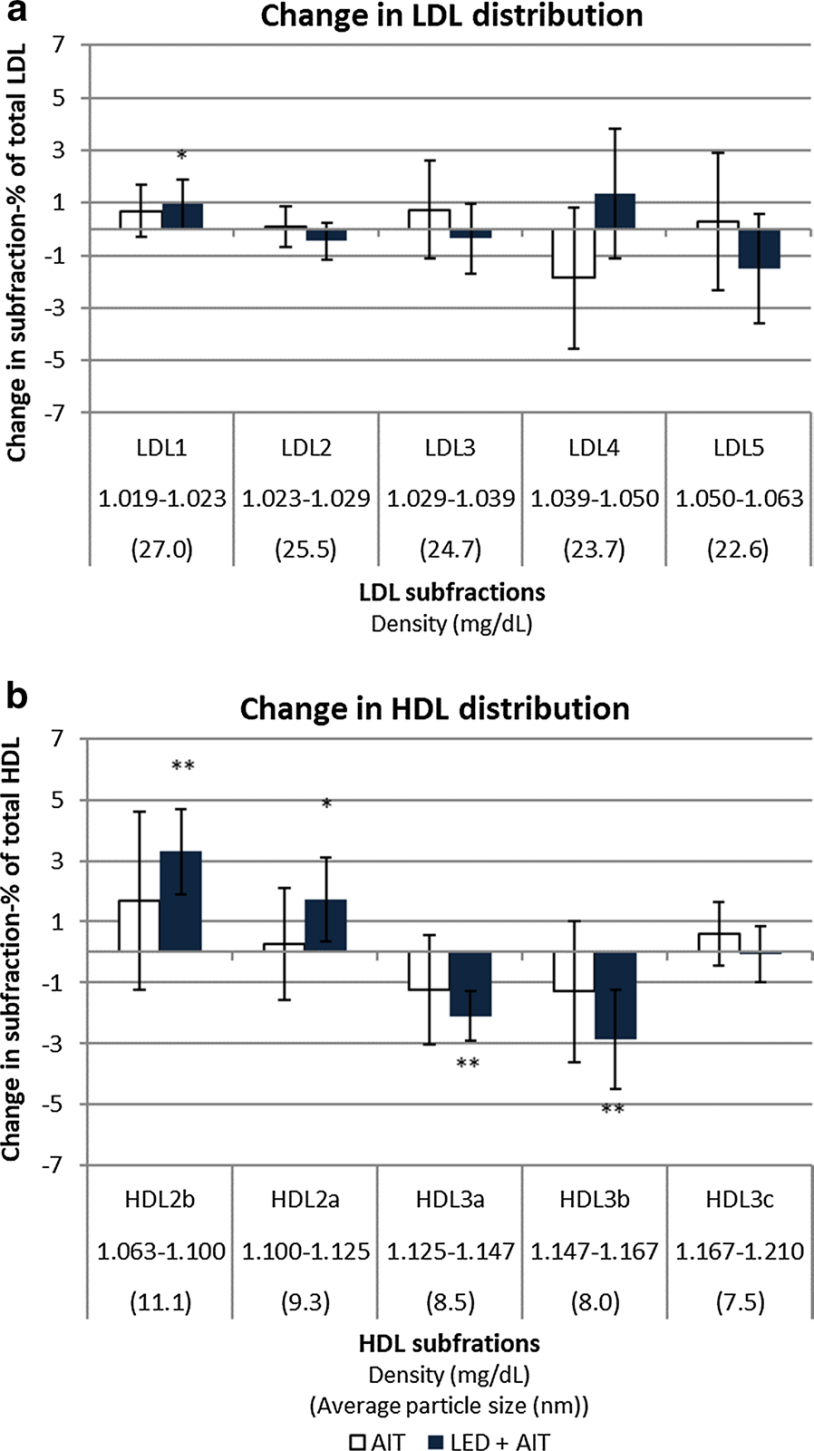
Change in the distribution of LDL and HDL from baseline to 1 year. **a** Changes baseline to 1 year in the distribution of LDL-subfractions by the change in the proportion that each subfraction constitutes of total LDL. **b** Changes in the distribution of HDL-subfractions by the change in the proportion that each subfraction constitutes of total HDL. Error-bars: 95% CI. Within-group difference: *p < 0.05, ***p ≤ 0.001. No between-group differences were seen. *AIT* aerobic interval training, *LED* low energy diet, *LDL* low density lipoprotein, *HDL* high density lipoprotein

Based on density profiling the 12-week intervention reduced AUC total lipoprotein and LDL in both groups while a shift toward a less atherogenic lipid profile was only seen after LED [28]. Decreases in total lipoprotein, LDL and HDL AUC in both groups as well as reductions in small, dense LDL AUC following weight loss were not maintained after 1 year. Reduced triglyceride-rich lipoprotein persisted only in the LED + AIT group (Fig. 2a, Additional file 1: Table S5). After 1 year LED + AIT but not AIT, showed increased HDL particle size and induced a shift in the HDL distribution to increase the proportion of large HDL subclasses, HDL_2a_ and HDL_2b_ (Fig. 2b). Furthermore, the contribution of HDL to total lipoprotein AUC increased following LED + AIT (baseline: 56.4%, difference: 2.5% (0.7%; 4.3%) p = 0.005). However, no significant between-group differences were seen at 1 year. All participants had LDL pattern B (LDL particle size ≤ 25 Å) [40] both before and after the intervention. At 1 year there was no difference between the intention to treat and per protocol analyses (Additional file 1: Table S6).

### Inflammatory markers

After 12 weeks there was largely no effect on inflammatory markers in either group except a decrease in TNFα following weight loss [28]. After 1 year the AIT group obtained a significant 35% (p = 0.019) decrease in CRP while TNFα and suPAR remained unchanged. Intention-to-treat analyses showed a significant decrease following LED + AIT in TNFα, suPAR and CRP of 13% (p < 0.001), 11% (p < 0.001) and 33% (p = 0.040), respectively. In the per protocol analyses the 31% decrease in CRP following AIT + LED was statistically non-significant (p = 0.081). This was possibly due to a smaller sample size. After 1 year a significant between-group difference was seen regarding suPAR (p = 0.036) (Additional file 1: Tables S7 and S8).

### Blood pressure and HADS

We observed no changes in blood pressure after 12 weeks’ intervention but there is a possible trend towards a reduction of these values. Moreover, six participants had their antihypertensive treatment reduced following LED [29]. No significant changes were seen in blood pressure after the one-year intervention. Excluding four patients with changes in their medication did not affect the conclusion. Following the 12-week intervention a slight improvement was seen in HADS-A [29] while no overall significant changes was seen after 1 year in neither HADS-A nor HADS-D (data not shown).

## Discussion

In this randomised study we compared a combination of weight loss and interval training with interval training alone during a 1-year intervention in overweight, sedentary CAD patients. The main findings were a similar improvement in exercise capacity in the two groups while the combined intervention achieved superior improvements in body composition. After 1 year both groups obtained a decrease in total cholesterol, non-HDL cholesterol and triglycerides while total cholesterol/HDL ratio was only decreased after LED + AIT. The effects on LDL particle size and atherogenicity were limited in both groups; however, a shift toward larger more buoyant HDL particles and an increase in the proportion of total lipoprotein constituted by HDL was seen following LED + AIT. Low-grade inflammation as indicated by CRP was reduced in both groups but TNFα and suPAR were only reduced in the LED + AIT group.

A recent survival analyses including participants without cardiovascular disease examine the interplay between characteristics of physical activity and cardiovascular disease prevention in nine subgroups divided by cardiovascular risk based on e.g. insulin resistance, hypertension, weight and dyslipidaemia. They conclude that physical activity with increased energy expenditure is associated with cardiovascular disease prevention but only in the low-risk subgroups. Hence, the beneficial effect of physical activity in high-risk subgroups is attenuated. However, the registered physical activity level was low in the high-risk subgroups overall making generalisability difficult [41]. Previous interventional studies addressing lifestyle changes have mainly included healthy individuals and excluded participants with pre-existing CAD or CVD. The current trial includes overweight, sedentary individuals with CAD and insulin resistance resembling the population described in the EUROASPIRE cohort [5].

A Cochrane review found that exercise-based cardiac rehabilitation programmes lower hospital admissions, total and cardiovascular mortality [4] and observational studies show that impaired peak aerobic capacity is a strong predictor of mortality in CAD patients [42]. Few interventional studies have described the effect of lifestyle changes on hard cardiovascular end-points. However, the 2013 the Look AHEAD trial that include 5145 participants with type 2 diabetes, showed that despite an improved cardiovascular risk profile and glycaemic control there was no difference in cardiovascular mortality between usual care and intensive lifestyle change after a median follow-up period of 9.6 years. Notably, the participants undergoing lifestyle changes had difficulty sustaining the initial improvements obtained [43]. The current trial was not designed or powered to address hard cardiovascular end-points.

### Physical fitness and body composition

Our findings on physical fitness agree with two earlier studies. A study from 2011 included 93 healthy, obese participants undergoing 1 year of either exercise, diet induced weight loss or a combined diet and exercise intervention [30]. A smaller 2006 study compared 6 months’ combined exercise and weight loss to a no therapy control group [44, 45]. In both of those studies the combined interventions improved physical performance and VO_2_peak (mL/kg/min). In the trial from 2011 the combined intervention was superior to the other interventional groups with within-group differences similar to the present trial. In interventional studies marked changes in body weight introduce a bias when using VO_2_peak_bw_ to describe physical fitness. Therefore, the values for VO_2_peak adjusted for body weight reported in 2011 may have been overestimated by lack of adjustment for the greater weight loss [30]. We calculated VO_2_peak_ffm_ (mL/kg fat free mass^0.67^/min) because energy expenditure during exercise is primarily in muscle tissue and VO_2_peak_ffm_ provides a fitness estimate that is independent of body weight [36]. Using this approach the LED + AIT intervention still elicited a larger increase in physical fitness at 1 year; however, there was no significant difference between the groups on any of the physical fitness variables (Table 2). Based on the iPAQ questionnaires the LED + AIT group had a greater increase in their weekly energy expenditure possibly explaining the increase in VO_2_peak despite a lower training attendance than the AIT group. However, the iPAQ results are subject to some uncertainty as reflected by the large confidence intervals; moreover, there was no between-group difference after 1 year when correcting for baseline values.

The improvements in body composition observed in the current trial correspond well to those reported in the two interventional trials cited above [30, 45] except for significant losses of fat free mass after the combined weight loss and exercise interventions. Nonetheless, in the 2011 trial the loss of fat free mass induced by the combined diet and exercise intervention was smaller than in the diet group (1.8 and 3.2 kg, respectively). In a randomised trial an approximate 10 kg weight loss induced by either caloric restriction or exercise only the exercise group maintained lower extremity muscle size and absolute strength [46]. Even though the loss of fat free mass was limited after LED in the present trial [29], weight loss interventions without exercise introduce a risk of losing lean body mass. This is an important consideration when designing cardiac rehabilitation programme, since low lean body mass is related to increased mortality in CAD [47]. A recent systematic review and meta-analysis showed that healthy individuals with the metabolic syndrome obtain improvements in body composition, metabolic and cardiovascular risk factors like those seen after AIT in the present trial [25]. Even though exercise alone only leads to a small weight loss a concomitant decreases in waist circumference is often seen as in the present trial [25]. Abdominal obesity is related to increased mortality in CAD [3]. In the current trial we showed a significant decrease in waist circumference and visceral abdominal fat after 12 weeks’ AIT [29]. The decrease in waist circumference persisted after 1 year (Table 2). It is well-described that visceral adipose tissue is linked to insulin resistance, dyslipidaemia and low-grade inflammation [48]. In addition, a recent Norwegian study show that 12 weeks’ exercise elicit a normalisation of macrophage-related mRNA transcript levels in subcutaneous white adipose tissue. This was closely related to improved insulin sensitivity in overweight, sedentary, dysglycaemic men and suggest that subcutaneous adipose tissue could also be an important mediator of exercise-induced decreases in the inflammatory response and improvements in insulin sensitivity delaying the development of type 2 diabetes [49].

### Lipids and density profiling

After 1 year a significant decrease in chemically determined total cholesterol, non-HDL-C and triglycerides was observed in both groups while LDL-C and HDL-C remained unchanged. A decrease in total cholesterol/HDL-C-ratio was only seen after LED + AIT. Density profiling was used to provide additional information on lipoprotein particle density and subclass distribution. The main finding was a shift in HDL subclass distribution and increased HDL particle size in the LED + AIT group while no changes were seen following AIT alone. The limited effect seen following exercise differs from outcomes from a recent meta-analysis comprising six trials, with a total of 1555 participants without CAD and 10 different exercise interventions [18]. That analysis found that exercise resulted in a shift toward larger LDL and HDL particles. The total amount of exercise in our trial was possibly insufficient to achieve similar effects on particle size. Overall, the exercise interventions in the meta-analysis lasted 20–35 weeks, the intensities were generally lower than in the current trial while the weekly amount of time spent exercising was higher. Additionally, some methodological differences may occur, since particle size in the meta-analyses was determined using nuclear magnetic resonance spectroscopy, while it was calculated from density distributions in the current trial. In the STRRIDE study, participants who underwent 8 months’ high-amount of high-intensity exercise training obtained a shift toward larger HDL and LDL particles while the effects of a low amount of high-intensity exercise were less pronounced [19]. Two previous studies comparing weight loss induced by either exercise or diet also found varying effects on LDL and HDL levels and particle size [16, 17]. As in the current trial, the populations in those studies were small (15–47 participants in each group) and some of the discrepancies could be related to sample size. In the present trial all participants exhibited a preponderance of small dense LDL particles, the so called LDL pattern B throughout the study [40]. The inability to maintain the improvements in LDL distribution and particle size obtained after LED could be due to return to neutral or even positive energy balance. Furthermore, the participants in LED + AIT group stabilised their BMI at ~ 30 kg/m^2^. An interventional trial including 100 participants showed that BMI < 25 kg/m^2^ was required to convert LDL pattern B to LDL pattern A [50] suggesting that the weight loss in the present trial may not have been sufficient. Finally, participants in the present study were contemporarily treated with statins. Since statins have been shown to decrease LDL levels and increase HDL and LDL particle size this could attenuate the effect of the intervention compared with a healthy, statin naïve population [51, 52].

### Inflammatory markers

During the first 12 weeks changes in markers of low-grade inflammation was limited in both groups [28]. However, more distinct effects on CRP, TNFα and suPAR were seen after 1 year. In observational studies elevated levels of CRP, TNFα and suPAR were associated with a poor prognosis in CAD [13–15] suggesting that the decreases in low-grade inflammation obtained in the present study might improve the participants’ prognosis. The reduced CRP-levels following 1 year of AIT corresponds well to a meta-analysis including 23 interventional studies demonstrating that exercise training is associated with reduced CRP-levels in CAD patients [21]. The lack of effect on TNFα after 1 year of AIT agrees with a study of 12 patients with ischaemic heart failure undergoing 4 months’ exercise training that showed no change in TNFα [53]. However, soluble TNF1- and TNF2-receptors were decreased significantly in these same subjects suggesting an attenuated inflammatory response. Moreover, an eight-week exercise-based cardiac rehabilitation programme elicited no reduction in neither TNFα nor CRP in 96 CAD patients despite improved VO_2_peak corrected for body weight [20]. Our findings that CRP was reduced 1 year of AIT suggests that duration of exercise is important. In the present trial CRP did not change significantly after the first 12 weeks’ exercise [28] and a small Russian study that compared 8 weeks’ moderate intensity training with high intensity interval training in overweight and obese young adults even found an increase in CRP following high intensity exercise [54]. An acute increase in CRP has been seen immediately after exercise [55]; however, in the Russian trial blood samples were drawn > 48 h after the last exercise session [54]. Few studies have described the effects of a long-term intervention and the studies included in the meta-analyses cited above had only 3–24 weeks’ exercise and lifestyle intervention [21]. Regarding weight loss our results are supported by a review comprising 33 interventional studies concluding that CRP is reduced after weight loss in a healthy population [22]. In the DIOGenes trial the group undergoing 8 weeks’ of LED followed by 26 weeks’ high protein, low glycaemic load diet obtained a decrease in CRP similar to our results [56]. The concurrent decrease in TNFα and suPAR following LED + AIT suggests an overall decrease in low-grade inflammation.

Notably, there is a significant between-group difference in suPAR levels after 1 year. Studies addressing the effects of lifestyle interventions on suPAR levels are sparse; however, a recent randomised trial including 133 healthy participants undergoing 5 months’ exercise intervention obtained no effect on suPAR levels supporting our findings [57]. In addition, observational data in 5538 individuals showed no relationship between self-reported leisure-time physical activity and suPAR levels when adjusting for lifestyle and cardiovascular risk factors [58]. In both studies an association between BMI and suPAR levels was seen [57, 58] possibly explaining the effect of weight loss, but not exercise, on suPAR levels. However, this conclusion is not certain since another observational study of 2273 individuals without cardiovascular disease reported it findings. That study found that suPAR was only positively related to BMI and waist circumference in female smokers, while a negative association was seen in non-smoking men and women [59]. Thus, further studies are needed to establish the relationship between physical activity, body weight and suPAR.

### Strengths and limitations

This randomised trial addresses both the effects of an intensive lifestyle intervention as published previously [28, 29] and the participants ability to maintain the improvements achieved in physical fitness and metabolic risk as presented in the current paper. Both interventions were feasible, and the participants were well-monitored by dieticians and physiotherapists. However, when applying the results to rehabilitation programmes selection bias and generalisability must be considered. The enrolled participants could be more motivated to engage in lifestyle changes than the CAD population in general. Transferring the results to individuals with co-morbidities covered by the exclusion criteria e.g. diabetes, chronic obstructive pulmonary disease and severe heart failure should be done carefully.

Drop-out rates (26% and 17% in the AIT and LED + AIT group, respectively) imply that intensive lifestyle changes require physical and mental strength and support from relatives and employers especially when considering long-term interventions. In addition, eight participants in the LED + AIT group followed the maintenance diet but did not participate in the exercise training. Our main analysis included all participants who attended the baseline visit and both follow-up visits irrespective of adherence to protocol. However, drop-out rates introduce a risk of bias due to small sample size and challenges related to generalisability as discussed above. Sample size calculations showed that 26 participants were required in each group; thus anticipating 25% drop-out we included 70 participants [35].

Despite randomisation a significant baseline difference regarding previous MI was seen. However, all participants had stable CAD diagnosed > 6 months prior to inclusion. The groups were similar regarding symptoms, risk factors, medication and left ventricular ejection fraction (Table 1) and are considered comparable.

## Conclusion

In this sedentary, overweight population with CAD, dyslipidaemia and insulin resistance the interventions resulted in similar improvements in physical fitness, serum-lipids and inflammation. The combination of diet and exercise as an intervention was superior in achieving long-term improvements in body composition. This suggests that a programme including both caloric restriction and exercise can be recommended in CAD rehabilitation to reduce cardiovascular and metabolic risk factors.

## Supplementary information

**Additional file 1.** Additional Figure and Tables.

## Data Availability

The datasets generated during and/or analysed during the current study are not publicly available due to directions from the Danish Dataprotection Agency but are available from the corresponding author on reasonable request.

## Abbreviations

AIT: aerobic interval training
BMI: body mass index
CRP: C-reactive protein
CPET: cardiopulmonary exercise test
HDL: high-density lipoprotein
LDL: low-density lipoprotein
LED: low energy diet
suPAR: soluble urokinase plasminogen activator receptor
TNFα: tumour necrosis factor α
TRL: triglyceride-rich lipoprotein
VO_2_peak: peak aerobic capacity
VO_2_peak_total_: total peak aerobic capacity (mL/min)
VO_2_peak_bw_: peak aerobic capacity corrected for body weight (mL/kg body weight/min)
VO_2_peak_ffm_: peak aerobic capacity corrected for fat free mass (mL/kg fat free mass^0.67^/min)
