# Supplementary material for: Effects of 1 year of exercise training versus combined exercise training and weight loss on body composition, low-grade inflammation and lipids in overweight patients with coronary artery disease: a randomized trial
Source: Cardiovasc Diabetol. 2019 Oct 1;18:127. doi: 10.1186/s12933-019-0934-x (PMC6774219; doi:10.1186/s12933-019-0934-x)
Supplement: Supplementary file 1 — Additional file 1. Additional Figure and Tables. [file 12933_2019_934_MOESM1_ESM.docx]

# Additional online material

## Figure S1:


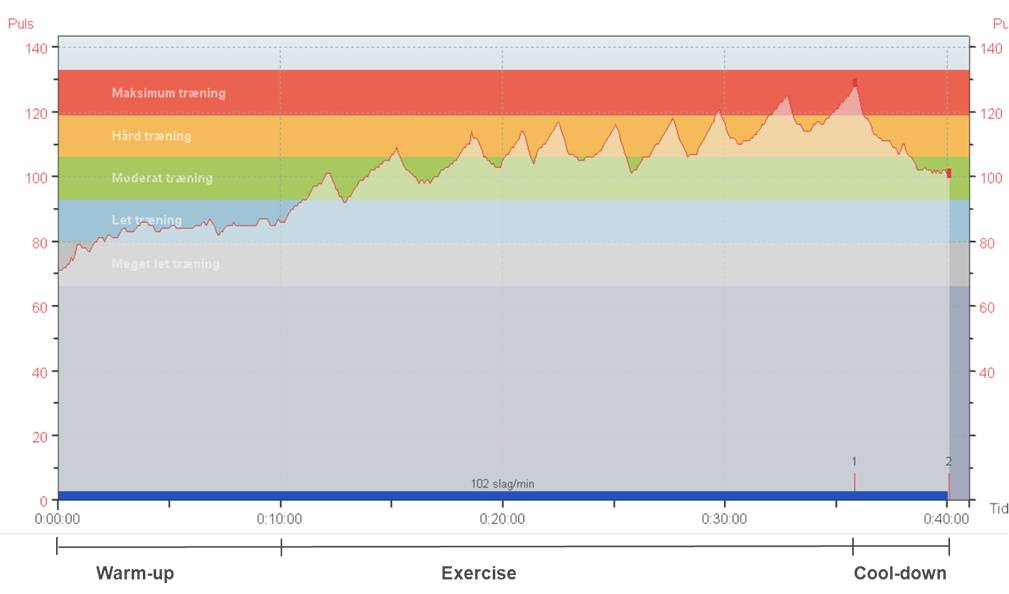


Heart rate curve. Heart rate is shown on the y-axis and time is shown on the x-axis. The coloured zones represent percentage of peak heart rate: Red: 90-100%, yellow: 80-90%, green: 70-80% and blue 60-70%.

## **Table S1: Baseline data of the per protocol population**

|  | **AIT (n=25)** | **LED+AIT (n=26)** | **P** |
| --- | --- | --- | --- |
| Male | 21 (84%) | 18 (69%) | 0.32 |
| Age | 62.2 (5.8) | 63.8 (7.1) | 0.40 |
| VO_2_peak_ffm_ (mL/kg ffm^0.67^/min) | 124 (25) | 124 (25) | 0.94 |
| VO_2_peak_bw_ (mL/kg/min) | 20.8 (5.0) | 20.6 (5.1) | 0.87 |
| Body weight (kg) | 96.1 (14.1) | 94.1 (9.8) | 0.56 |
| Body mass index (kg/m^2^) | 31.5 (29.6;33.5) | 31.2 (29.9;32.7) | 0.92 |
| Body fat mass (kg) | 32.8 (7.7) | 33.7 (7.5) | 0.64 |
| Waist circumference (cm) | 110 (9.7) | 107 (7.3) | 0.33 |
| Hip circumference (cm) | 111 (8.2) | 110 (7.4) | 0.62 |
| Systolic blood pressure (mmHg) | 126 (13) | 128 (15) | 0.76 |
| Diastolic blood pressure (mmHg) | 74 (9.3) | 70 (7.4) | 0.10 |
| Total cholesterol (mmol/L) | 4.3 (0.8) | 4.1 (0.7) | 0.23 |
| Left ventricular ejection fraction (%) | 53 (8) | 53 (7) | 0.92 |
| **Ischaemic aetiology and treatment** | | | |
| Myocardial infarction | 8 (32%) | 19 (73%) | 0.005 |
| Percutaneous Coronary Intervention | 16 (64%) | 20 (77%) | 0.37 |
| Coronary artery bypass graft | 6 (24%) | 6 (23%) | 1.00 |
| Prior cardiac rehabilitation | 23 (92%) | 18 (69%) | 0.08 |
| **CCS-class** | | | |
| 0 | 21 (84%) | 21 (81%) |  |
| I | 4 (16%) | 5 (19%) | 0.89 |
| **NYHA-class** | | | |
| I | 20 (80%) | 19 (73%) |  |
| II | 5 (20%) | 6 (23%) | 0.58 |
| III | 0 (0%) | 1 (4%) |  |
| **Medication** | | | |
| ACE-I/ARB | 14 (56%) | 17 (65%) | 0.78 |
| Acetylsalisylic acid | 22 (88%) | 23 (88%) | 1.00 |
| Beta blocker | 12 (48%) | 14 (54%) | 0.78 |
| Calcium antagonist | 6 (24%) | 10 (38%) | 0.39 |
| Statin | 24 (96%) | 25 (96%) | 1.00 |
| Other cholesterol-lowering drug | 5 (20%) | 2 (8%) | 0.25 |

Baseline characteristics per protocol population. Categorical data: number (%), normally distributed data: mean (SD), non-normally distributed data: median (IQ-range). P-values: between-group differences.

AIT, Aerobic interval training, LED, Low energy diet, VO_2_peak_bw_, Peak aerobic capacity corrected for body weight, VO_2_peak_ffm_, Peak aerobic capacity corrected for fat free mass, CCS, Canadian Cardiovascular Society, NYHA, New York Heart Association, ACE-I, Angiotensin Converting Enzyme Inhibitor, ARB, Angiotensin receptor blocker.

## Table S2: Body composition and physical fitness. Per protocol

|  | AIT (n=25) | | | | | LED + AIT (n=26) | | | | | Between-group  1 year* | Interaction between group and time |
| --- | --- | --- | --- | --- | --- | --- | --- | --- | --- | --- | --- | --- |
|  | Baseline | Change  Baseline to 1 year | P | Change  12 week to  1 year | P | Baseline | Change  Baseline to 1 year | P | Change  12 week to 1 year | P |  | P |
| Body composition | | | | | | | | | | | | |
| Weight (kg) | 96.1  (14.1) | -1.7  (-2.7;-0.7) | 0.001 | -0.5  (-1.5;0.5) | 0.348 | 94.1 (9.8) | -7.8  (-8.2;-6.7) | <0.001 | 2.5  (1.4;3.6) | <0.001 | 5.7  (4.1;7.3) | <0.001 |
| Body mass index (kg/m^2^) | 32.2  (3.2) | -0.5  (-0.9;-0.2) | 0.001 | -0.2  (-0.5;0.2) | 0.358 | 32.1 (3.1) | -2.7  (-3.0;-2.3) | <0.001 | 0.9  (0.5;1.2) | <0.001 | 2.1  (1.6;2.6) | <0.001 |
| Body fat mass (kg) | 32.8  (7.7) | -1.9  (-2.9;-1.0) | <0.001 | -0.3  (-1.3;0.6) | 0.454 | 33.8 (7.5) | -7.1  (-8.2;-5.9) | <0.001 | 2.0  (0.9;3.2) | <0.001 | 5.1  (3.7;6.6) | <0.001 |
| Body fat % | 34.8  (6.5) | -1.5  (-2.4;-0.7) | <0.001 | -0.2  (-1.0;0.7) | 0.657 | 36.6 (6.9) | -5.1  (-6.2;-4.0) | <0.001 | 1.8  (0.7;2.9) | 0.001 | 3.6  (2.2;5.0) | <0.001 |
| Fat free mass (kg) | 62.5  (9.9) | 0.2  (-0.5;0.8) | 0.646 | -0.3  (-1.0;0.3) | 0.330 | 59.7 (8.8) | -0.6  (-1.2;0.002) | 0.049 | 0.3  (-0.3;0.9) | 0.382 | 0.8  (-0.1;1.7) | 0.099 |
| Waist (cm) | 109.8 (9.7) | -3.4  (-5.2;-1.6) | <0.001 | -0.7  (-2.5;1.2) | 0.475 | 107.4 (7.3) | -7.1  (-9.2;-5.0) | <0.001 | 3.4  (1.4;5.5) | 0.001 | 3.7  (0.9;6.5) | 0.010 |
| Hip (cm) | 110.8 (8.2) | -3.9  (-5.1;-2.8) | <0.001 | -1.7  (-2.8;-0.5) | 0.005 | 109.7 (7.4) | -6.5  (-7.9;-5.0) | <0.001 | 0.9  (-0.5;2.3) | 0.221 | 2.5  (0.7;4.4) | 0.008 |
| Waist/hip-ratio | 0.99 (0.07) | 0.003  (-0.01;0.02) | 0.652 | 0.009  (-0.001;0.02) | 0.255 | 0.98 (0.07) | -0.008  (-0.03;0.01) | 0.399 | 0.03  (0.01;0.04) | 0.007 | 0.01  (-0.01;0.04) | 0.354 |
| Physical fitness | | | | | | | | | | | | |
| VO_2_peak_total_ (mL/min) | 1989 (471) | 105  (-24;235) | 0.111 | -117  (-247;12) | 0.076 | 1947 (531) | 124  (19;228) | 0.020 | 192  (88;297) | <0.001 | -18.6  (-184.8;147.6) | 0.826 |
| VO_2_peak_kg_ (mL//kg/min) | 20.8 (5.0) | 1.6  (0.2;3.0) | 0.021 | -0.9  (-2.3;0.5) | 0.203 | 20.6 (5.1) | 3.2  (2.0;4.4) | <0.001 | 1.5  (0.3;2.7) | 0.017 | -1.6  (-3.4;0.3) | 0.101 |
| VO_2_peak_ffm_  (mL/kg fat free mass^0.67^/min) | 123.9 (24.8) | 6.5  (-1.5;14.6) | 0.112 | -7.0  (-15.0;1.1) | 0.091 | 124.5 (25.5) | 8.3  (1.8;14.8) | 0.012 | 11.8  (5.3;18.3) | <0.001 | -1.8  (-12.1;8.6) | 0.739 |
| Peak RER | 1.2 (0.1) | -0.01  (-0.04;0.02) | 0.595 | 0.01  (-0.02;0.04) | 0.485 | 1.2 (0.1) | 0.1  (0.04;0.1) | <0.001 | 0.01 | 0.569 | 0.1  (0.03;0.1) | 0.001 |
| Max workload (watt) | 158 (43) | 11  (1;22) | 0.032 | -7  (-17;4) | 0.192 | 153 (49) | 15  (8;22) | <0.001 | 15  (8;22) | <0.001 | -4 (-16;9) | 0.549 |
| Peak heart rate (beats/min) | 138 (23) | 1  (-4;7) | 0.606 | 0.3  (-6;5) | 0.918 | 134 (23) | 7  (3;12) | <0.001 | 8  (4;13) | <0.001 | -6  (0.8;13) | 0.086 |

Baseline: mean (SD) Baseline data are presented as mean (SD), within- and between-group differences are presented with 95% C.I. AIT, Aerobic interval training, LED, Low energy diet, VO_2_peak_total_, Total peak aerobic capacity, VO_2_peak_bw_, Peak aerobic capacity corrected for body weight, VO_2_peak_ffm_, Peak aerobic capacity corrected for fat free mass, CPET, Cardiopulmonary exercise test, RER, respiratory exchange ratio. *Between-group difference at 1 year corrected for baseline difference

## Table S3: Lipids, intention-to-treat

|  | AIT (n=25) | | | | | LED + AIT (n=28) | | | | | Between-group  1 year* | Interaction between group and time |
| --- | --- | --- | --- | --- | --- | --- | --- | --- | --- | --- | --- | --- |
|  | Baseline | Change  Baseline to 1 year | P | Change  12 week to 1 year | P | Baseline | Change  Baseline to 1 year | P | Change  12 week to 1 year | P |  | P |
| Total cholesterol (mmol/L) | 4.3 (0.8) | -0.3  (-0.6;-0.05) | 0.019 | 0.02  (-0.2;0.3) | 0.870 | 4.1 (0.7) | -0.4  (-0.6;-0.2) | 0.001 | 0.1  (-0.2;0.3) | 0.480 | 0.1  (-0.2;0.4) | 0.564 |
| LDL (mmol/L) | 2.3 (0.5) | -0.1  (-0.3;0.05) | 0.138 | 0.1  (-0.1;0.3) | 0.598 | 2.2 (0.6) | -0.2  (-0.3;0.04) | 0.116 | 0.1  (-0.1;0.3) | 0.536 | 0.002  (-0.3;0.3) | 0.987 |
| Non-HDL (mmol/L) | 3.1 (0.6) | -0.3  (-0.5;-0.1) | 0.014 | 0.05  (-0.2;0.3) | 0.679 | 2.9 (0.7) | -0.4  (-0.6;-0.2) | <0.001 | 0.05  (-0.2;0.2) | 0.656 | 0.1  (-0.2;0.4) | 0.479 |
| HDL (mmol/L) | 1.2 (0.3) | -0.02  (-0.1;0.1) | 0.740 | -0.03  (-0.1;0.1) | 0.586 | 1.1 (0.2) | -0.01  (-0.1;0.1) | 0.847 | 0.04  (-0.03;0.1) | 0.278 | -0.01  (-0.1;0.1) | 0.887 |
| Total cholesterol / HDL-C | 3.6 (0.7) | -0.2  (-0.2;0.1) | 0.256 | 0.1  (-0.2;0.4) | 0.449 | 3.7 (0.8) | -0.3  (-0.5;-0.1) | 0.003 | -0.03  (-0.2;0.2) | 0.762 | 0.1  (-0.2;0.5) | 0.389 |
| Triglycerides | 1.9 (1.2) | -0.4  (-0.7;-0.04) | 0.027 | 0.01  (-0.4;0.3) | 0.954 | 1.6 (0.7) | -0.5  (-0.7;-0.4) | <0.001 | -0.02  (-0.2;0.1) | 0.786 | 0.1  (-0.3;0.5) | 0.531 |

Baseline: mean (SD) Baseline data are presented as mean (SD), within- and between-group differences are presented with 95% C.I. AIT, Aerobic interval training, LED, Low energy diet, LDL, low density lipoprotein, HDL, high-density lipoprotein. *Between-group difference at 1 year corrected for baseline difference

## Table S4: Lipids, per protocol

|  | AIT (n=24) | | | | | LED + AIT (n=25) | | | | | Between-group  1 year* | Interaction between group and time |
| --- | --- | --- | --- | --- | --- | --- | --- | --- | --- | --- | --- | --- |
|  | Baseline | Change  Baseline to 1 year | P | Change  12 weeks to 1 year | P | Baseline | Change  Baseline to 1 year | P | Change  12 weeks to 1 year | P |  | P |
| Total cholesterol (mmol/L) | 4.4 (0.8) | -0.3  (-0.5;0.03) | 0.031 | 0.01  (-0.2;0.3) | 0.920 | 4.2 (0.7) | -0.4  (-0.7;-0.2) | 0.001 | 0.1  (-0.2;0.3) | 0.480 | 0.1  (-0.2;0.5) | 0.513 |
| LDL-C (mmol/L) | 2.3 (0.5) | -0.1  (-0.3;0.1) | 0.181 | 0.04  (-0.2;0.2) | 0.714 | 2.3 (0.6) | -0.2  (-0.4;0.05) | 0.126 | 0.1  (-0.2;0.3) | 0.580 | 0.02  (-0.3;0.3) | 0.875 |
| Non-HDL-C (mmol/L) | 3.1 (0.6) | -0.3  (-0.5;-0.04) | 0.023 | 0.04  (-0.2;0.3) | 0.754 | 3.0 (0.7) | -0.4  (-0.6;-0.2) | <0.001 | 0.1  (-0.2;0,3) | 0.647 | 0.2  (-0.2;0.5) | 0.348 |
| HDL-C (mmol/L) | 1.2 (0.3) | -0.01  (-0.1;0.1) | 0.803 | -0.02  (-0.1;0.1) | 0.643 | 1.2 (0.2) | 0.02  (-0.1;0.1) | 0.602 | 0.04  (-0.03;0.1) | 0.261 | -0.03  (-0.2;0.1) | 0.598 |
| Total cholesterol / HDL-C | 3.6 (0.7) | -0.1  (-0.4;0.1) | 0.299 | 0.1  (-0.2;0.4) | 0.546 | 3.7 (0.8) | -0.4  (-0.6;-0.2) | <0.001 | -0.03  (-0.2;02) | 0.753 | 0.2  (-0.1;0.6) | 0.171 |
| Triglycerides | 1.9 (1.3) | -0.4  (-0.8;-0.03) | 0.035 | 0.01  (-0.4;0.4) | 0.943 | 1.6 (0.7) | -0.6  (-0.7;-0.4) | <0.001 | -0.01  (-0.2;0.2) | 0.912 | 0.2  (-0.2;0.6) | 0.426 |

Baseline: mean (SD) Baseline data are presented as mean (SD), within- and between-group differences are presented with 95% C.I. AIT, Aerobic interval training, LED, Low energy diet, LDL, low density lipoprotein, HDL, high-density lipoprotein. *Between-group difference at 1 year corrected for baseline difference

## Table S5: Density profiling, intention-to-treat

|  | AIT (n=26) | | | | | LED + AIT (n=29) | | | | | Between-group  1 year* | Interaction between group and time |
| --- | --- | --- | --- | --- | --- | --- | --- | --- | --- | --- | --- | --- |
|  | Baseline | Change  Baseline to 1 year | P | Change  12 week to 1 year | P | Baseline | Change  Baseline to 1 year | P | Change  12 week to 1 year | P |  | P |
| TRL | 420 (231) | -19  (-108;70) | 0.679 | 74  (-16;164) | 0.109 | 433 (254) | -138  (-223;-53) | 0.002 | 6  (-79;91) | 0.890 | 117  (-6;240) | 0.062 |
| ***LDL*** | | | | | | | | | | | | |
| LDL_1_ | 65 (34) | 7  (-12;26) | 0.447 | 12  (-7;32) | 0.210 | 57 (30) | 10  (-2;22) | 0.114 | 14  (-2;-26) | 0.026 | -2  (-25;20) | 0.818 |
| LDL_2_ | 108 (42) | -6  (-22;11) | 0.488 | 17  (0.5;34) | 0.044 | 100 (45) | -7  (-21;7) | 0.322 | 13  (-0.4;27) | 0.058 | 1  (-20;22) | 0.914 |
| LDL_3_ | 274 (77) | 5  (-39;49) | 0.833 | 41  (-4;85) | 0.072 | 271 (98) | 3  (-31;37) | 0.856 | 33  (-0.9;67) | 0.056 | 1  (-56;54) | 0.984 |
| LDL_4_ | 617 (246) | -40  (-112;33) | 0.287 | 42  (-32;116) | 0.267 | 576 (223) | 36  (-34;106) | 0.314 | 136  (66;206) | <0.001 | -74  (-176;27) | 0.152 |
| LDL_5_ | 445 (172) | -0.6  (-76;75) | 0.987 | 63  (-13;139) | 0.106 | 391 (168) | -24 (-75;26) | 0.341 | 107  (57;157) | <0.001 | 23  (-65;112) | 0.603 |
| Total LDL | 1511 (414) | -32  (-210;147) | 0.730 | 175  (-6;356) | 0.058 | 1396 (391) | 19  (-119;157) | 0.787 | 305  (167;443) | <0.001 | -51  (-274;173) | 0.657 |
| ***HDL*** | | | | | | | | | | | | |
| HDL_2b_ | 593 (278) | 54  (-52;161) | 0.319 | 86  (-22;194) | 0.119 | 496 (177) | 108  (46;170) | 0.001 | 123  (61;185) | <0.001 | 53  (-173;67) | 0.387 |
| HDL_2a_ | 560 (241) | 2  (-74;77) | 0.965 | 96  (20;173) | 0.014 | 492 (136) | 79  (16;141) | 0.014 | 136  (73;198) | <0.001 | -76  (174;22) | 0.127 |
| HDL_3a_ | 721 (222) | -41  (-119;36) | 0.295 | 70  (-8;148) | 0.080 | 687 (135) | -14  (-67;38) | 0.590 | 120  (68;172) | <0.001 | -27  (65;118) | 0.565 |
| HDL_3b_ | 461 (146) | -36  (-99;27) | 0.261 | 22  (-85;41) | 0.497 | 467 (126) | -58  (-100;-15) | 0.008 | 52  (10;95) | 0.016 | 22  (-52;96) | 0.565 |
| HDL_3c_ | 174 (48) | 15  (-8;39) | 0.202 | 11  (-13;35) | 0.368 | 181 (45) | 3  (-17;22)) | 0.795 | 41  (21;60) | <0.001 | 13  (-17;43) | 0.406 |
| Total HDL | 2509 (759) | -14  (241;213) | 0.904 | 229  (-0.6;460) | 0.051 | 2325 (481) | 117  (-52;286) | 0.173 | 472  (303;641) | <0.001 | -130  (-409;149) | 0.362 |
| Total lipoprotein | 4440 (1184) | -63  (-455;328) | 0.751 | 480  (83;877) | 0.018 | 4155 (916) | 2  (-326;331) | 0.989 | 787  (458;1116) | <0.001 | -63  (-571;445) | 0.809 |
| Particle size | | | | | | | | | | | | |
| LDL diameter | 23.8 (0.24) | 0.03  (-0.2;0.08) | 0.232 | 0.04  (-0.01;0.1) | 0.132 | 23.9 (0.15) | 0.04  (-0.01;0.1) | 0.099 | 0.06  (-0.1;0.01) | 0.011 | -0.01  (-0.1;0.1) | 0.848 |
| HDL diameter | 9.1 (0.22) | 0.05  (-0.04;0.1) | 0.317 | 0.09  (0.001;0.2) | 0.048 | 9.0 (0.2) | 0.11  (0.1;0.2) | <0.001 | 0.02  (-0.04;0.1) | 0.585 | -0.1  (-0.2;0.04) | 0.210 |

Baseline data are presented as mean (SD), within- and between-group differences are presented with 95% C.I. AIT, Aerobic interval training, LED, Low energy diet, TRL, Triglyceride-rich lipoprotein, LDL, Low-density lipoprotein, HDL, High-density lipoprotein.

## Table S6: Density profiling, per protocol

|  | AIT (n=24) | | | | | LED + AIT (n=25) | | | | | Between-group  1 year* | Interaction between group and time |
| --- | --- | --- | --- | --- | --- | --- | --- | --- | --- | --- | --- | --- |
|  | Baseline | Change  Baseline to 1 year | P | Change  12 week to 1 year | P | Baseline | Change  Baseline to 1 year | P | Change  12 week to 1 year | P |  | P |
| TRL | 423 (235) | -31  (-122;60) | 0.507 | 61  (-31;154) | 0.191 | 451 (263) | -165  (-254;76) | <0.001 | 5  (-84;94) | 0.913 | 132  (5;258) | 0.041 |
| LDL | | | | | | | | | | | | |
| LDL_1_ | 66  (35) | 7  (-13;27) | 0.488 | 11  (-9;31) | 0.282 | 58  (30) | 9  (-5;23) | 0.195 | 14  (-0.03;56) | 0.050 | -2  (-26;22) | 0.858 |
| LDL_2_ | 109  (43) | -6  (-23;11) | 0.500 | 16  (-1;33) | 0.070 | 99  (44) | -9  (-24;5) | 0.201 | 12  (-2;27) | 0.097 | 4  (-18;26) | 0.748 |
| LDL_3_ | 277  (78) | 7  (-39;53) | 0.769 | 42  (-4;88) | 0.077 | 275  (98) | -7  (-42;29) | 0.711 | 27  (-8;63) | 0.131 | 12  (-45;70) | 0.676 |
| LDL_4_ | 613 (250) | -34  (-109;41) | 0.376 | 41  (-35;118) | 0.291 | 592 (224) | 26  (-49;102) | 0.494 | 137  (62;214) | <0.001 | -59  (-165;48) | 0.281 |
| LDL_5_ | 447 (176) | 2  (-76;80) | 0.959 | 61  (-18;140) | 0.130 | 394 (173) | -30  (-84;25) | 0.290 | 115 (60;169) | <0.001 | 31  (-63;126) | 0.514 |
| Total LDL | 1511 (423) | -22 (-207) | 0.818 | 171  (-16;259) | 0.074 | 1418 (380) | -8  (-155;139) | 0.912 | 307  (161;454) | <0.001 | -13  (-248;222) | 0.912 |
| HDL | | | | | | | | | | | | |
| HDL_2b_ | 601 (280) | 56  (-55;167) | 0.325 | 86  (-27;199) | 0.134 | 501 (186) | 113 (45;181) | 0.001 | 130 (62;197) | <0.001 | -57  (-185;72) | 0.387 |
| HDL_2a_ | 565 (245) | 7  (-71;86) | 0.860 | 98 (19;178) | 0.015 | 502 (141) | 78  (10;145) | 0.025 | 142 (74;210) | <0.001 | -70  (-173;34) | 0.187 |
| HDL_3a_ | 723 (226) | -39  (-119;42) | 0.346 | 71  (-11;152) | 0.090 | 699 (136) | -25 (-80;30) | 0.377 | 120 (65;176) | <0.001 | -14  (-111;83) | 0.783 |
| HDL_3b_ | 464 (148) | -43  (-108;21) | 0.186 | -23  (-89;42) | 0.479 | 477  (127) | -72  (-118;-27) | 0.002 | 42  (-4;87) | 0.074 | 29  (-49;107) | 0.467 |
| HDL_3c_ | 175  (48) | 13  (-11;37) | 0.290 | 12  (-12;36) | 0.340 | 185  (45) | -4 (-25;17) | 0.699 | 34  (14;55) | 0.001 | 17  (-14;49) | 0.286 |
| Total HDL | 2529 (768) | -14  (-251;222) | 0.906 | 232  (-8;472) | 0.058 | 2365 (489) | 90  (-91;271) | 0.331 | 468 (288;649) | <0.001 | -102  (-193;399) | 0.497 |
| Total | 4462 (1102) | -65  (-473;342) | 0.753 | 467 (53;879) | 0.027 | 4234 (921) | -79  (-430;271) | 0.657 | 785  (435;1135) | <0.001 | 17  (-519;553) | 0.951 |
| Particle size | | | | | | | | | | | | |
| LDL diameter (nm) | 23.8 (0.2) | 0.03  (-0.02;0.1) | 0.172 | 0.04  (-0.01;0.1) | 0.280 | 23.9 (0.2) | 0.03  (-0.02;0.1) | 0.187 | -0.1  (-0.1;0.03) | 0.003 | -0.003  (-0.1;0.1) | 0.942 |
| HDL diameter (nm) | 9.1  (0.2) | 0.05  (-0.04;0.2) | 0.276 | 0.09  (-0.003;0.2) | 0.057 | 9.0  (0.2) | 0.13  (0.1;0.2) | <0.001 | 0.03  (-0.03;0.1) | 0.314 | -0.1  (-0.2;0.03) | 0.162 |

Baseline: mean (SD), within- and between-group differences are presented with 95% C.I. AIT, Aerobic interval training, LED, Low energy diet, TRL, Triglyceride-rich lipoprotein, LDL, Low-density lipoprotein, HDL, High-density lipoprotein. *Between-group difference at 1 year corrected for baseline difference

## Table S7: Inflammatory markers. Intention-to-treat.

|  | AIT (n=25) | | | | | LED + AIT (n=28) | | | | | Interaction between group and time |
| --- | --- | --- | --- | --- | --- | --- | --- | --- | --- | --- | --- |
|  | Baseline | Change  Baseline to 1 year | P | Change  12 week to 1 year | P | Baseline | Change  Baseline to 1 year | P | Change  12 week to 1 year | P | P |
| CRP (mg/L) | 2.0 (0.8;3.9) | -35%  (-55%;-7%) | 0.019 | -25%  (-48%;8%) | 0.122 | 1.3  (0.7;3.5) | -33%  (-54%;-2%) | 0.040 | -3%  (-33%;42%) | 0.893 | 0.848 |
| TNFα (pg/mL) | 5.1 (4.7;7.1) | -7%  (-16%;3%) | 0.148 | -3%  (-12%;7%) | 0.530 | 6.1  (5.2;6.8) | -13%  (-18%;-7%) | <0.001 | -2%  (-8%;-5%) | 0.593 | 0.255 |
| suPAR (ng/mL) | 4.7 (3.9;5.2) | -0.4%  (-7%;7%) | 0.905 | 1%  (-6%;9%) | 0.747 | 5.0  (4.1;5.9) | -11%  (-15%;-6%) | <0.001 | -8%  (-13%;3%) | 0.002 | 0.017 |

Baseline: Median (IQ-range). Change: Percentage change (95% C.I.). AIT, aerobic interval training, LED, low energy diet, CRP, C-reactive protein, TNFα, tumour necrosis factor α, suPAR, soluble urokinase plasminogen activator receptor.

## Table S8: Inflammatory markers. Per protocol.

|  | AIT (n=24) | | | | | LED + AIT (n=25) | | | | | Interaction between group and time |
| --- | --- | --- | --- | --- | --- | --- | --- | --- | --- | --- | --- |
|  | Baseline | Change  Baseline to 1 year | P | Change  12 week to 1 year | P | Baseline | Change  Baseline to 1 year | P | Change  12 week to 1 year | P | P |
| CRP (mg/L) | 2.1 (0.8;3.9) | -35%  (-55%;-5%) | 0.025 | -26%  (-49%;7%) | 0.113 | 1.1 (0.7;3.4) | -31%  (-55%;5%) | 0.081 | -1%  (-35%;50%) | 0.969 | 0.817 |
| TNFα (pg/mL) | 5.2 (4.8;7.1) | -7%  (-16%;3%) | 0.152 | -3%  (-12;8%) | 0.571 | 5.8 (5.0;6.8) | -12%  (-17%;-6%) | <0.001 | -3%  (-9%;3%) | 0.341 | 0.370 |
| suPAR (ng/mL) | 4.7 (3.9;5.2) | -1%  (-8%;7%) | 0.787 | 1%  (-6%;9%) | 0.757 | 5.0 (4.1;5.9) | -12%  (-16%;-6%) | <0.001 | -10%  (-15%;-4%) | 0.001 | 0.023 |

Baseline: Median (IQ range). Change: Percentage change (95% C.I.).

AIT, aerobic interval training, LED, low energy diet, CRP, C-reactive protein, TNFα, tumour necrosis factor α, suPAR, soluble urokinase plasminogen activator receptor.
